# *catena*-Poly[[[(triphenyl­phosphane)copper(I)]-di-μ-iodido-[(triphenyl­phosphane)copper(I)]-μ-[3,6-bis­(4-pyrid­yl)-1,2,4,5-tetra­zine]] acetonitrile disolvate]

**DOI:** 10.1107/S1600536810042807

**Published:** 2010-10-30

**Authors:** Jinfang Zhang

**Affiliations:** a Molecular Materials Research Center, Scientific Research Academy, School of Chemistry and Chemical Engineering, Jiangsu University, Zhenjiang 212013, People’s Republic of China

## Abstract

The title compound, {[Cu_2_I_2_(C_12_H_8_N_6_)(C_18_H_15_P)_2_]·2CH_3_CN}_*n*_, contains centrosymmetric dinuclear Cu_2_I_2_(PPh_3_)_2_ units bridged by 3,6-bis­(4-pyrid­yl)-1,2,4,5-tetra­zine ligands lying also across crystallographic inversion centers, giving a chain structure in the *ab* plane. The distorted tetra­hedral Cu^I^ atoms in the dinuclear unit are coordinated by two bridging iodide anions, one pyridine N atom from the substituted tetra­zine ligand and one terminal triphenyl­phosphine P-atom donor. The Cu⋯Cu distance is 2.8293 (12) Å, implying a weak Cu⋯Cu inter­action.

## Related literature

For examples of metal-organic compounds with intriguing architectures and topologies, see: Eddaoudi *et al.* (2001 ▶). For potential applications of these compounds, see: Banerjee *et al.* (2008 ▶); Zhang *et al.* (2007 ▶). For examples of metal-organic frameworks constructed using long bridging ligands, see: Withersby *et al.* (2000 ▶).
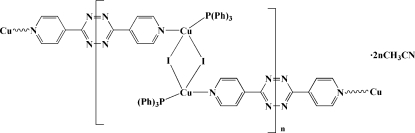


## Experimental

### 

#### Crystal data

[Cu_2_I_2_(C_12_H_8_N_6_)(C_18_H_15_P)_2_]·2C_2_H_3_N
*M* *_r_* = 1223.80
Monoclinic, 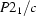

*a* = 12.344 (3) Å
*b* = 11.675 (2) Å
*c* = 18.521 (4) Å
β = 101.41 (3)°
*V* = 2616.4 (10) Å^3^
*Z* = 2
Mo *K*α radiation
μ = 2.10 mm^−1^
*T* = 293 K
0.25 × 0.20 × 0.15 mm

#### Data collection

Rigaku Saturn724 diffractometer
Absorption correction: multi-scan (*CrystalClear*; Rigaku, 2007 ▶) *T*_min_ = 0.779, *T*_max_ = 1.000
12271 measured reflections
5042 independent reflections
4233 reflections with *I* > 2s*I*)
*R*_int_ = 0.030

#### Refinement

*R*[*F* ^2^ > 2σ(*F* ^2^)] = 0.040
*wR*(*F* ^2^) = 0.079
*S* = 1.09
5042 reflections
299 parameters
H-atom parameters constrained
Δρ_max_ = 0.49 e Å^−3^
Δρ_min_ = −0.47 e Å^−3^

### 

Data collection: *CrystalClear* (Rigaku, 2007 ▶); cell refinement: *CrystalClear*; data reduction: *CrystalClear* ; program(s) used to solve structure: *SHELXS97* (Sheldrick, 2008 ▶); program(s) used to refine structure: *SHELXL97* (Sheldrick, 2008 ▶); molecular graphics: *SHELXTL* (Sheldrick, 2008 ▶); software used to prepare material for publication: *SHELXTL*.

## Supplementary Material

Crystal structure: contains datablocks I, global. DOI: 10.1107/S1600536810042807/zs2071sup1.cif

Structure factors: contains datablocks I. DOI: 10.1107/S1600536810042807/zs2071Isup2.hkl

Additional supplementary materials:  crystallographic information; 3D view; checkCIF report

## Figures and Tables

**Fig. 1.**
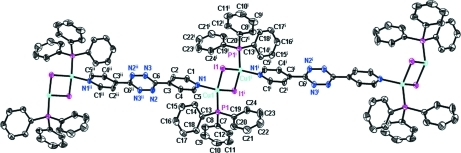
The molecular structure of a portion of the title compound, with atom labels and 30% probability displacement ellipsoids. All H atoms have been omitted. Symmetry codes: (i) -x, -y, -z; (ii) -x + 1, -y - 1, -z.

## supplementary crystallographic information

### Comment

Metal-organic frameworks have attracted great attention in recent years not only because of their intriguing structures (Eddaoudi *et al.*, 2001) but also their potential applications (Banerjee *et al.*, 2008; Zhang *et al.*, 2007). Long bridging ligands can be employed for the construction of interesting metal-organic frameworks (Withersby *et al.*, 2000). The extended bridging ligand 2,6-bis(4-pyridyl)-1,2,4,5-tetrazine was used to synthesize the title compound {[Cu_2_I_2_(C_12_H_8_N_6_)((C_6_H_5_)_3_P)_2_ .2(CH_3_CN)}*_n_* (3,6-di_2_(PPh_3_)_2_ (I) by using a diffusion reaction and the crystal structure is presented here.

The centrosymmetric dinuclear Cu_2_I_2_(PPh_3_)_2_ complex units in (I) are linked by the extended 3,6-di-4-pyridyl-1,2,4,5-tetrazine ligands, also lying across crystallographic inversion centers, giving a one-dimensional chain structure (Fig. 1). Each tetrahedral Cu^I^ centre in the dinuclear unit is coordinated by two bridging I anions [Cu—I, 2.6412 (9), 2.6603 (9) Å], one pyridine-N from the bridging substituted tetrazine ligand [Cu—N, 2.066 (3)Å] and one terminal triphenylphosphine P-donor [Cu—P, 2.2388 (11) Å]. The Cu···Cu^i^ distance is 2.8293 (12) Å, implying a weak Cu···Cu interaction [symmetry code: (i) -*x*, -*y*, -*z*].

### Experimental

CuI (0.1 mmol) and triphenylphosphine (0.2 mmol) were added to a mixture of 3 ml of dimethylformamide and 2 ml of H_3_CN with thorough stirring for 2 minutes. After filtering, the filtrate was carefully layered with a solution of 0.1 mmol 3,6-bis(4-pyridyl)-1,2,4,5-tetrazine in 3 ml of CH_2_Cl_2_. Blue block crystals were obtained after two weeks.

### Refinement

H atoms were positioned geometrically with C—H(phenyl, pyridyl) = 0.93 Å or 0.96 Å (methyl) and refined using a riding model, with *U*_iso_(H) = 1.2*U*_eq_(C)_phenyl, pyridyl_ or 1.5*U*_eq_(C)_methyl_.

### Crystal data

**Table:**

[Cu_2_I_2_(C_12_H_8_N_6_)(C_18_H_15_P)_2_]·2C_2_H_3_N	*F*(000) = 1212
*M**_r_* = 1223.80	*D*_x_ = 1.553 Mg m^−^^3^
Monoclinic, *P*2_1_/*c*	Mo *K*α radiation, λ = 0.71073 Å
Hall symbol: -P 2ybc	Cell parameters from 10225 reflections
*a* = 12.344 (3) Å	θ = 2.8–29.1°
*b* = 11.675 (2) Å	µ = 2.10 mm^−^^1^
*c* = 18.521 (4) Å	*T* = 293 K
β = 101.41 (3)°	Block, blue
*V* = 2616.4 (10) Å^3^	0.25 × 0.20 × 0.15 mm
*Z* = 2	

### Data collection

**Table:**

Rigaku Saturn724 diffractometer	5042 independent reflections
Radiation source: fine-focus sealed tube	4233 reflections with *I* > 2s˘*I*)
graphite	*R*_int_ = 0.030
ω scans	θ_max_ = 26.0°, θ_min_ = 2.8°
Absorption correction: multi-scan (*CrystalClear*; Rigaku, 2007)	*h* = −15→11
*T*_min_ = 0.779, *T*_max_ = 1.000	*k* = −14→14
12271 measured reflections	*l* = −18→22

### Refinement

**Table:**

Refinement on *F*^2^	Primary atom site location: structure-invariant direct methods
Least-squares matrix: full	Secondary atom site location: difference Fourier map
*R*[*F*^2^ > 2σ(*F*^2^)] = 0.040	Hydrogen site location: inferred from neighbouring sites
*wR*(*F*^2^) = 0.079	H-atom parameters constrained
*S* = 1.09	*w* = 1/[σ^2^(*F*_o_^2^) + (0.0303*P*)^2^ + 0.5456*P*] where *P* = (*F*_o_^2^ + 2*F*_c_^2^)/3
5042 reflections	(Δ/σ)_max_ = 0.001
299 parameters	Δρ_max_ = 0.49 e Å^−^^3^
0 restraints	Δρ_min_ = −0.47 e Å^−^^3^

### Special details

**Table:**

Geometry. All e.s.d.'s (except the e.s.d. in the dihedral angle between two l.s. planes) are estimated using the full covariance matrix. The cell e.s.d.'s are taken into account individually in the estimation of e.s.d.'s in distances, angles and torsion angles; correlations between e.s.d.'s in cell parameters are only used when they are defined by crystal symmetry. An approximate (isotropic) treatment of cell e.s.d.'s is used for estimating e.s.d.'s involving l.s. planes.
Refinement. Refinement of *F*^2^ against ALL reflections. The weighted *R*-factor *wR* and goodness of fit *S* are based on *F*^2^, conventional *R*-factors *R* are based on *F*, with *F* set to zero for negative *F*^2^. The threshold expression of *F*^2^ > σ(*F*^2^) is used only for calculating *R*-factors(gt) *etc*. and is not relevant to the choice of reflections for refinement. *R*-factors based on *F*^2^ are statistically about twice as large as those based on *F*, and *R*- factors based on ALL data will be even larger.

### Fractional atomic coordinates and isotropic or equivalent isotropic displacement parameters (Å^2^)

**Table:**

	*x*	*y*	*z*	*U*_iso_*/*U*_eq_	
I1	−0.01591 (2)	−0.05190 (2)	0.113971 (13)	0.04604 (10)	
Cu1	0.11635 (4)	0.00036 (4)	0.02284 (2)	0.04082 (13)	
P1	0.23134 (8)	0.14551 (8)	0.06241 (5)	0.0398 (2)	
N1	0.1998 (2)	−0.1497 (2)	0.01109 (16)	0.0407 (7)	
N2	0.4794 (3)	−0.4221 (3)	−0.05567 (17)	0.0466 (8)	
N3	0.4453 (3)	−0.4974 (3)	0.05724 (16)	0.0468 (8)	
N4	0.1913 (5)	0.7319 (6)	0.2563 (3)	0.121 (2)	
C6	0.4276 (3)	−0.4218 (3)	0.0010 (2)	0.0393 (9)	
C13	0.3435 (3)	0.1093 (3)	0.13879 (19)	0.0433 (9)	
C2	0.3008 (3)	−0.3135 (3)	0.0653 (2)	0.0518 (10)	
H2	0.3187	−0.3623	0.1055	0.062*	
C23	0.0102 (5)	0.3983 (5)	0.0741 (4)	0.101 (2)	
H23	−0.0614	0.4153	0.0498	0.121*	
C22	0.0648 (6)	0.4706 (5)	0.1276 (4)	0.106 (2)	
H22	0.0300	0.5367	0.1394	0.128*	
C21	0.1706 (5)	0.4459 (4)	0.1637 (4)	0.0941 (19)	
H21	0.2070	0.4945	0.2004	0.113*	
C3	0.3460 (3)	−0.3303 (3)	0.00372 (19)	0.0383 (8)	
C1	0.2290 (3)	−0.2235 (3)	0.0664 (2)	0.0517 (10)	
H1	0.1990	−0.2137	0.1083	0.062*	
C8	0.3772 (4)	0.1219 (4)	−0.0334 (2)	0.0648 (12)	
H8	0.3975	0.0542	−0.0078	0.078*	
C7	0.3016 (3)	0.1947 (3)	−0.01016 (19)	0.0463 (9)	
C4	0.3140 (3)	−0.2564 (3)	−0.0553 (2)	0.0483 (10)	
H4	0.3409	−0.2660	−0.0984	0.058*	
C19	0.1689 (3)	0.2757 (3)	0.0911 (2)	0.0481 (10)	
C16	0.5102 (4)	0.0355 (4)	0.2532 (2)	0.0654 (13)	
H16	0.5662	0.0099	0.2910	0.079*	
C5	0.2420 (3)	−0.1687 (3)	−0.0490 (2)	0.0472 (10)	
H5	0.2214	−0.1197	−0.0890	0.057*	
C17	0.5309 (4)	0.1236 (4)	0.2086 (2)	0.0631 (12)	
H17	0.5998	0.1587	0.2166	0.076*	
C18	0.4468 (3)	0.1597 (4)	0.1513 (2)	0.0554 (11)	
H18	0.4606	0.2190	0.1208	0.066*	
C14	0.3247 (4)	0.0209 (3)	0.1853 (2)	0.0522 (10)	
H14	0.2557	−0.0141	0.1779	0.063*	
C24	0.0620 (4)	0.2999 (4)	0.0565 (3)	0.0708 (13)	
H24	0.0245	0.2500	0.0210	0.085*	
C11	0.3177 (5)	0.3187 (5)	−0.1108 (3)	0.0841 (16)	
H11	0.2973	0.3857	−0.1371	0.101*	
C15	0.4076 (4)	−0.0155 (4)	0.2426 (2)	0.0646 (13)	
H15	0.3940	−0.0740	0.2737	0.078*	
C10	0.3914 (5)	0.2462 (6)	−0.1326 (3)	0.0908 (19)	
H10	0.4206	0.2632	−0.1740	0.109*	
C12	0.2727 (4)	0.2936 (4)	−0.0497 (2)	0.0648 (12)	
H12	0.2226	0.3440	−0.0353	0.078*	
C20	0.2222 (4)	0.3491 (4)	0.1454 (3)	0.0706 (13)	
H20	0.2939	0.3326	0.1697	0.085*	
C9	0.4224 (4)	0.1483 (5)	−0.0934 (3)	0.0834 (16)	
H9	0.4741	0.0994	−0.1075	0.100*	
C26	−0.0159 (6)	0.7161 (6)	0.2552 (3)	0.125 (3)	
H26A	−0.0565	0.7385	0.2076	0.187*	
H26B	−0.0362	0.7646	0.2922	0.187*	
H26C	−0.0329	0.6380	0.2647	0.187*	
C25	0.1023 (7)	0.7271 (6)	0.2569 (3)	0.0907 (19)	

### Atomic displacement parameters (Å^2^)

**Table:**

	*U*^11^	*U*^22^	*U*^33^	*U*^12^	*U*^13^	*U*^23^
I1	0.04230 (17)	0.05492 (18)	0.04190 (15)	0.00885 (12)	0.01074 (11)	0.00954 (12)
Cu1	0.0381 (3)	0.0371 (3)	0.0471 (3)	0.0054 (2)	0.0081 (2)	0.0014 (2)
P1	0.0371 (5)	0.0416 (5)	0.0413 (5)	−0.0004 (4)	0.0089 (4)	0.0018 (4)
N1	0.0381 (18)	0.0412 (17)	0.0443 (17)	0.0114 (14)	0.0116 (14)	0.0046 (15)
N2	0.048 (2)	0.0468 (18)	0.0485 (18)	0.0197 (16)	0.0180 (15)	0.0066 (15)
N3	0.050 (2)	0.0467 (18)	0.0454 (18)	0.0190 (16)	0.0146 (15)	0.0055 (16)
N4	0.108 (5)	0.154 (5)	0.095 (4)	0.031 (5)	0.005 (4)	0.022 (3)
C6	0.037 (2)	0.0367 (19)	0.044 (2)	0.0082 (16)	0.0076 (17)	0.0012 (17)
C13	0.037 (2)	0.052 (2)	0.0395 (19)	0.0020 (18)	0.0045 (17)	−0.0006 (18)
C2	0.062 (3)	0.049 (2)	0.049 (2)	0.027 (2)	0.019 (2)	0.0137 (19)
C23	0.076 (4)	0.080 (4)	0.138 (5)	0.033 (3)	−0.001 (4)	−0.028 (4)
C22	0.103 (5)	0.062 (3)	0.158 (6)	0.020 (3)	0.033 (5)	−0.038 (4)
C21	0.082 (4)	0.072 (4)	0.129 (5)	−0.004 (3)	0.023 (4)	−0.046 (3)
C3	0.033 (2)	0.037 (2)	0.045 (2)	0.0086 (16)	0.0087 (16)	−0.0022 (17)
C1	0.056 (3)	0.056 (2)	0.049 (2)	0.025 (2)	0.0236 (19)	0.012 (2)
C8	0.066 (3)	0.070 (3)	0.062 (3)	−0.005 (3)	0.022 (2)	−0.003 (2)
C7	0.045 (2)	0.053 (2)	0.041 (2)	−0.0093 (19)	0.0079 (18)	0.0023 (19)
C4	0.051 (2)	0.053 (2)	0.044 (2)	0.019 (2)	0.0167 (18)	0.0094 (19)
C19	0.047 (2)	0.044 (2)	0.055 (2)	−0.0004 (19)	0.0153 (19)	−0.002 (2)
C16	0.060 (3)	0.085 (3)	0.046 (2)	0.014 (3)	−0.004 (2)	−0.010 (2)
C5	0.046 (2)	0.052 (2)	0.045 (2)	0.0205 (19)	0.0113 (18)	0.0115 (19)
C17	0.044 (3)	0.082 (3)	0.060 (3)	−0.006 (2)	0.002 (2)	−0.006 (3)
C18	0.046 (3)	0.068 (3)	0.052 (2)	−0.002 (2)	0.008 (2)	0.002 (2)
C14	0.052 (3)	0.060 (3)	0.045 (2)	−0.001 (2)	0.009 (2)	0.002 (2)
C24	0.060 (3)	0.063 (3)	0.084 (3)	0.012 (2)	0.002 (3)	−0.014 (3)
C11	0.095 (4)	0.095 (4)	0.062 (3)	−0.029 (3)	0.014 (3)	0.023 (3)
C15	0.078 (4)	0.066 (3)	0.046 (2)	0.009 (3)	0.003 (2)	0.009 (2)
C10	0.102 (5)	0.129 (5)	0.048 (3)	−0.047 (4)	0.032 (3)	−0.002 (3)
C12	0.068 (3)	0.066 (3)	0.061 (3)	−0.011 (2)	0.011 (2)	0.014 (2)
C20	0.055 (3)	0.063 (3)	0.094 (3)	−0.007 (2)	0.015 (3)	−0.028 (3)
C9	0.086 (4)	0.102 (4)	0.074 (3)	−0.020 (3)	0.044 (3)	−0.021 (3)
C26	0.147 (7)	0.118 (5)	0.129 (6)	0.040 (5)	0.076 (5)	0.056 (4)
C25	0.118 (6)	0.096 (4)	0.060 (3)	0.033 (5)	0.022 (4)	0.022 (3)

### Geometric parameters (Å, °)

**Table:**

Cu1—N1	2.066 (3)	C8—C7	1.392 (6)
Cu1—P1	2.2388 (11)	C8—H8	0.9300
Cu1—I1^i^	2.6603 (9)	C7—C12	1.376 (6)
Cu1—Cu1^i^	2.8293 (12)	C4—C5	1.376 (5)
P1—C13	1.823 (4)	C4—H4	0.9300
P1—C19	1.830 (4)	C19—C24	1.378 (5)
P1—C7	1.831 (4)	C19—C20	1.384 (6)
N1—C1	1.333 (4)	C16—C17	1.374 (6)
N1—C5	1.338 (4)	C16—C15	1.378 (6)
N2—N3^ii^	1.327 (4)	C16—H16	0.9300
N2—C6	1.333 (5)	C5—H5	0.9300
N3—N2^ii^	1.327 (4)	C17—C18	1.395 (5)
N3—C6	1.349 (4)	C17—H17	0.9300
N4—C25	1.102 (8)	C18—H18	0.9300
C6—C3	1.476 (5)	C14—C15	1.386 (6)
C13—C18	1.382 (5)	C14—H14	0.9300
C13—C14	1.393 (5)	C24—H24	0.9300
C2—C1	1.378 (5)	C11—C10	1.361 (8)
C2—C3	1.378 (5)	C11—C12	1.387 (6)
C2—H2	0.9300	C11—H11	0.9300
C23—C22	1.372 (8)	C15—H15	0.9300
C23—C24	1.385 (6)	C10—C9	1.368 (7)
C23—H23	0.9300	C10—H10	0.9300
C22—C21	1.376 (8)	C12—H12	0.9300
C22—H22	0.9300	C20—H20	0.9300
C21—C20	1.373 (6)	C9—H9	0.9300
C21—H21	0.9300	C26—C25	1.460 (9)
C3—C4	1.387 (5)	C26—H26A	0.9600
C1—H1	0.9300	C26—H26B	0.9600
C8—C9	1.374 (6)	C26—H26C	0.9600
			
Cu1—I1—Cu1^i^	64.51 (3)	C8—C7—P1	118.6 (3)
N1—Cu1—P1	112.29 (9)	C5—C4—C3	119.0 (4)
N1—Cu1—I1	104.81 (9)	C5—C4—H4	120.5
P1—Cu1—I1	113.43 (3)	C3—C4—H4	120.5
N1—Cu1—I1^i^	103.87 (8)	C24—C19—C20	119.0 (4)
P1—Cu1—I1^i^	106.64 (4)	C24—C19—P1	117.0 (3)
I1—Cu1—I1^i^	115.49 (3)	C20—C19—P1	123.9 (3)
N1—Cu1—Cu1^i^	117.65 (9)	C17—C16—C15	120.8 (4)
P1—Cu1—Cu1^i^	129.83 (4)	C17—C16—H16	119.6
I1—Cu1—Cu1^i^	58.07 (3)	C15—C16—H16	119.6
I1^i^—Cu1—Cu1^i^	57.42 (3)	N1—C5—C4	123.9 (3)
C13—P1—C19	105.43 (17)	N1—C5—H5	118.1
C13—P1—C7	104.18 (18)	C4—C5—H5	118.1
C19—P1—C7	103.94 (19)	C16—C17—C18	119.0 (4)
C13—P1—Cu1	114.42 (13)	C16—C17—H17	120.5
C19—P1—Cu1	116.59 (13)	C18—C17—H17	120.5
C7—P1—Cu1	111.05 (12)	C13—C18—C17	121.4 (4)
C1—N1—C5	116.3 (3)	C13—C18—H18	119.3
C1—N1—Cu1	122.1 (2)	C17—C18—H18	119.3
C5—N1—Cu1	120.8 (2)	C15—C14—C13	120.8 (4)
N3^ii^—N2—C6	117.8 (3)	C15—C14—H14	119.6
N2^ii^—N3—C6	117.0 (3)	C13—C14—H14	119.6
N2—C6—N3	125.2 (3)	C19—C24—C23	120.3 (5)
N2—C6—C3	117.7 (3)	C19—C24—H24	119.8
N3—C6—C3	117.0 (3)	C23—C24—H24	119.8
C18—C13—C14	118.3 (3)	C10—C11—C12	120.7 (5)
C18—C13—P1	124.4 (3)	C10—C11—H11	119.7
C14—C13—P1	117.3 (3)	C12—C11—H11	119.7
C1—C2—C3	119.3 (3)	C16—C15—C14	119.7 (4)
C1—C2—H2	120.4	C16—C15—H15	120.1
C3—C2—H2	120.4	C14—C15—H15	120.1
C22—C23—C24	119.8 (5)	C11—C10—C9	119.7 (5)
C22—C23—H23	120.1	C11—C10—H10	120.1
C24—C23—H23	120.1	C9—C10—H10	120.1
C23—C22—C21	120.4 (5)	C7—C12—C11	120.5 (5)
C23—C22—H22	119.8	C7—C12—H12	119.7
C21—C22—H22	119.8	C11—C12—H12	119.7
C20—C21—C22	119.7 (5)	C21—C20—C19	120.8 (5)
C20—C21—H21	120.2	C21—C20—H20	119.6
C22—C21—H21	120.2	C19—C20—H20	119.6
C2—C3—C4	117.6 (3)	C10—C9—C8	120.1 (5)
C2—C3—C6	121.5 (3)	C10—C9—H9	120.0
C4—C3—C6	120.9 (3)	C8—C9—H9	120.0
N1—C1—C2	123.9 (4)	C25—C26—H26A	109.5
N1—C1—H1	118.1	C25—C26—H26B	109.5
C2—C1—H1	118.1	H26A—C26—H26B	109.5
C9—C8—C7	121.1 (5)	C25—C26—H26C	109.5
C9—C8—H8	119.4	H26A—C26—H26C	109.5
C7—C8—H8	119.4	H26B—C26—H26C	109.5
C12—C7—C8	117.9 (4)	N4—C25—C26	177.2 (8)
C12—C7—P1	122.9 (3)		

Symmetry codes: (i) −*x*, −*y*, −*z*; (ii) −*x*+1, −*y*−1, −*z*.
